# New Simplified Diagnostic Decision Trees for the Detention of Metabolic Syndrome in the Elderly

**DOI:** 10.3390/ijerph17145191

**Published:** 2020-07-18

**Authors:** Enrique Rodríguez-Guerrero, Manuel Romero-Saldaña, Azahara Fernández-Carbonell, Rafael Molina-Luque, Guillermo Molina-Recio

**Affiliations:** 1 Lucena Health Center, Healthcare Management Area South of Córdoba, C/Paseo de Rojas No/No, 14900 Lucena, Spain; enriquerg83@gmail.com; 2 Department of Nursing, Faculty of Medicine and Nursing, University of Córdoba, Avd. Menéndez Pidal No/No, 14004 Córdoba, Spain; rafael.moluq@gmail.com (R.M.-L.); gmolina@uco.es (G.M.-R.); 3 Cardiovascular Surgery Service, Reina Sofía University Hospital, Avd. Menéndez Pidal No/No, 14004 Córdoba, Spain; azasa89@hotmail.com

**Keywords:** anthropometry, diagnosis, geriatrics, metabolic syndrome, primary care, rural health

## Abstract

Background: A new simplified method for the detention of metabolic syndrome (MetS) is proposed using two variables (anthropometric and minimally invasive). Methods: A study of MetS prevalence was made on a sample of 361 older people. The anthropometric variables analyzed were: blood pressure, body mass index, waist circumference (WC), waist–height ratio, body fat percentage, and waist–hip ratio. A crude and adjusted binary logistic regression was performed, and receiver operating characteristic curves were obtained for determining the predictive capacity of those variables. For the new detection method, decision trees were employed using automatic detection by interaction through Chi-square. Results: The prevalence of the MetS was of 43.7%. The final decision trees uses WC and basal glucose (BG), whose cutoff values were: for men, WC ≥ 102.5 cm and BG > 98 mg/dL (sensitivity = 67.1%, specificity = 90.3%, positive predictive value = 85%, validity index = 79.9%); and for women, WC ≥ 92.5 cm and BG ≥ 97 mg/dL (sensitivity = 65.9%, specificity = 92.7%, positive predictive value = 87.1%, validity index = 81.3%). In older women the best predictive value of MetS was a WC of 92.5 cm. Conclusions: It is possible to make a simplified diagnosis of MetS in older people using the WC and basal capillary glucose, with a high diagnostic accuracy and whose use could be recommended in the resource-poor health areas. A new cutting point in older women for the WC should be valued.

## 1. Introduction

The metabolic syndrome (MetS) is defined as being a group of risk factors characterized by central obesity, blood pressure, hyperglycemia, and alterations in the lipid metabolism, i.e., hypertriglyceridemia and a diminution in high-density cholesterol (c-HDL) [1]. The MetS is closely related to type 2 diabetes mellitus, cardiovascular diseases, and other cardiovascular risk factors that increase morbidity and mortality [2].

Nearly 50% of the elderly population in the USA suffers from MetS [3], while in the Asiatic old people population (*n* = 73,547), a prevalence of MetS of 42.6% has been observed [4]. According to the Spanish ENRICA study [5], the MetS prevalence in the elderly was 42.3%. This percentage is higher than other age groups, with 11.2% and 30.5% in those between 18 to 44 years and 45 to 64 years, respectively. For those reasons, the population made up of subjects of 65 years and over is probably one of the highest clinical interest for the development and application of non-invasive or minimally invasive methods that permit an early diagnosis of the MetS, especially in places where there is limited access to resources.

Given that prevalence, the diagnosis of MetS would facilitate a new prediction of cardiovascular events and total mortality beyond that supplied by other conventional risk factors [6]. Besides, the determination of abdominal obesity and, more specifically, visceral fat accumulation, has shown itself to be a critical factor in the pathogenesis of insulin resistance and the onset of the MetS [7,8]. Various studies [9,10,11] have demonstrated the real correlation between body and abdominal adiposity with anthropometric measurements like the body mass index (BMI), the waist circumference (WC), the waist–hip ratio (WHR), and the waist–height ratio (WHtR).

For all the above, the main objective of this research was to propose a non- or minimally invasive method for the detection of MetS in the elderly population.

## 2. Materials and Methods

### 2.1. Study Design. Population Sample

A prevalence study was carried out during 2019 on 361 older people. The reference population was formed by individuals of 65 years or over belonging to the Lucena Health Centre (Córdoba, Spain). Sampling was done randomly according to age and gender. The program EPIDAT version 4.2 (Department of Sanidade, Xunta de Galicia, Galicia, Spain) was used to calculate the sample size. For an elderly population of approximately 10,000 older people, an expected prevalence of 42.3% according to the ENRICA study [5], a 95% confidence level and a precision of 5%, the sample size obtained for the cross-sectional study was of 361 patients.

The inclusion criterion consisted of being 65 years of age or over, but excluding all those individuals with prolonged hospitalizations, with dementia, and with oncological diseases or palliative treatments.

### 2.2. Study Variables and Measurements

#### 2.2.1. Result Variable

It was the presence or not of MetS (dichotomic variable). Based on the harmonized definition [12], i.e., fulfilling three of the following five criteria: blood pressure (BP) ≥ 130/85 mmHg or being under antihypertensive treatment; triglycerides (TG) ≥ 150 mg/dL or under treatment with fenofibrate or nicotinic acid; c-HDL < 50 mg/dL for women and < 40 mg/dL for men, or under treatment with fenofibrate or nicotinic acid; fasting basal glucose ≥ 100 mg/dL, under hypoglycemic treatment or with a diagnosis of type 2 diabetes mellitus; WC ≥ 88 cm in women and ≥ 102 in men (European population cut-off points).

#### 2.2.2. Explanatory Variables (Independent Ones)

Personal and lifestyle variables: age, gender, educational level, tobacco use, alcohol consumption, physical activity performed (moderate–intense exercise > 30 min/day), daily consumption of fruit and vegetables, polymedication (taking five or more medicines a day during six months or more) [13].

Anthropometric and body composition variables: weight (kg), height (cm), WC (cm), BMI (kg/m^2^), waist–height ratio (WHtR): calculated by the ratio between the WC and height in centimeters; waist–hip ratio (WHR): calculated by the ratio between the WC and the hip circumference in centimeters; the percentage of body fat (BFP): calculated following the Deurenberg equation; ABSI (A BodyShapeIndex), BAI (BodyAdiposityIndex), systolic blood pressure (SBP) and diastolic blood pressure (DBP) expressed in mmHg.

Analysis variables: c-HDL (mg/dL), c-LDL (mg/dL), fasting plasma glucose (mg/dL) and triglycerides (mg/dL).

#### 2.2.3. Measurement

The data were collected through a medical consultation, where participants met with the principal investigator. The level of education was classified into no education, primary, secondary and university education. Concerning smoking habits, it was aimed to detect active, former, and non-smokers. The individuals were asked if they did a moderate–intense physical activity of ≥30 min/day and consumed fruit and vegetables daily, both questions taken from the FINRISK questionnaire [14]. Alcohol intake was also contemplated by following WHO recommendations adapted to Spain [15]. According to the latter, the levels considered to be of risk for persons of over 65 are ≥17 units of standard drinks per week or over 3 units of standard drink on one occasion. 

The anthropometric measurements (height, weight, waist and hip circumference) were taken following the recommendations in the reference manual for anthropometric standardization [16], and by trained and experienced staff, in order to minimize the coefficients of variation. The instructions issued by the European Society of Hypertension [17] to gauge the blood pressure were observed, using an automatic, calibrated sphygmomanometer (model Omron M3 comfort^®^, Kyoto, Japan). The BAI (Body Adiposity Index) [18] was obtained from the equation ((hip perimeter)/((Height)1.5)-18), and for the ABSI (A Body Shape Index) [19] the calculation was based on the WC, relating it to the BFP and the height. The analytical values like c-HDL, c-LDL, fasting plasma glucose and triglycerides were obtained from the last analysis made provided that it had been procured in under one year. If not, a new serological analysis was requested to determine more recent results.

### 2.3. Ethical Aspects

The Ethics Committee of the Andalusian Government approved this research project (Ethical code: File number 272, Reference 3711, Date of approval 02/01/2018). All the study individuals were informed verbally and in writing on the objectives of the health examination, with an informed consent being necessary under regulations in force. The study protocol obeyed the Declaration of Helsinki [20] to perform medical research with humans.

### 2.4. Statistical Analysis

For the statistical analysis, the programs IBM SPSS Statistics v.25.0 (IBM, Chicago, IL, USA) and EPIDAT version 4.2 (Department of Sanidade, Xunta de Galicia, Galicia, Spain) have been used. The continuous and discrete quantitative variables have been represented by the mean and standard deviation, whereas the qualitative or categorical variables were described by their absolute and relative frequency. To contrast the goodness of fit to a normal distribution of the data from continuous or discrete quantitative variables, when *n* > 50, the Kolmogorov–Smirnov test corrected by Lilliefors was used, and the graphic representations as histograms, graphs P-P and Q-Q; whereas, if *n* < 50, the Shapiro–Wilk test was applied. The homoscedasticity of variances was contrasted with the Levene test. For the comparison of two independent arithmetical means, the Student-t or Mann–Whitney U-tests were employed, as was indicated. For the comparison of three or more independent means, the ANOVA or Kruskal–Wallis tests were applied, according to whether or not it was a parametric test. A post hoc analysis through the Bonferroni and Tukey tests was made. The comparison of percentages was carried by the chi-square test, applying Fisher’s exact test when indicated. For contingency tables with ordinal variables, the Chi-squared of Mantel-Haensel, and the Somers d, the Kendall Tau-b and Tau-c tests were calculated. A multivariate analysis was performed through a crude and adjusted binary logistic regression, calculating the goodness of fit of the model from the deviance and the Hosmer–Lemeshow test. The Wald test was applied as a contrast statistic.

In addition, the crude odds ratio (OR) was calculated with 95% confidence intervals. Receiver Operating Characteristic curves (ROC) were made, and the Area Under the Curve (AUC) was reckoned in order to determine what explanatory variables best predicted the presence of MetS. The cut-off points for each predictive variable were established according to the Youden index (higher joint sensitivity and specificity). For the diagnostic test study, sensitivity, specificity, predictive values, likelihood ratios, Youden indices, and diagnostic validity or validity index were taken. Diverse clinical decision trees were set up (classification), employing for this purpose as a growth method the CHAID (Chi-squared Automatic Interaction Detection) technique. The level of statistical significance for the division of the nodes and the fusion of categories was *p* <0.05; the significance values were corrected by the Bonferroni method, and the number of maximum iterations was 100. Statistical significance was fixed for an alpha error of below 5%, and the confidence intervals were calculated with a 95% certainty.

## 3. Results

### 3.1. Prevalence of the Metabolic Syndrome

Of the 361 elderly individuals studied, 192 were women (53.2%). The mean age was of 73.2 ± 6.4 years (CI 95% 72.6–73.9 years). A total of 158 people had the MetS, with a prevalence of 43.8% (CI 95% 38.6–49.1%). The prevalence of MetS distinguished by gender was 42.7% (CI 95% 35.6–50%) and 45% (CI 95% 37.3–52.8%) for women and men, respectively. None of the personal or lifestyle variables gave statistically significant results, except for fruit and vegetable consumption, which was revealed as being a protective factor (OR = 0.44, CI 95% 0.27–0.72, *p* < 0.01); being polymedicated behaved as a risk factor (OR = 2.33, CI 95% 1.43–3.78, *p* < 0.01). Regarding the anthropometric variables, most of them were associated significantly with MetS except DBP.

Table 1 shows characteristics about the presence of MetS, or not, and a logistic regression with a crude OR.

### 3.2. Anthropometric Variables and Predictive Analyses of the MetS

**Figure 1:**
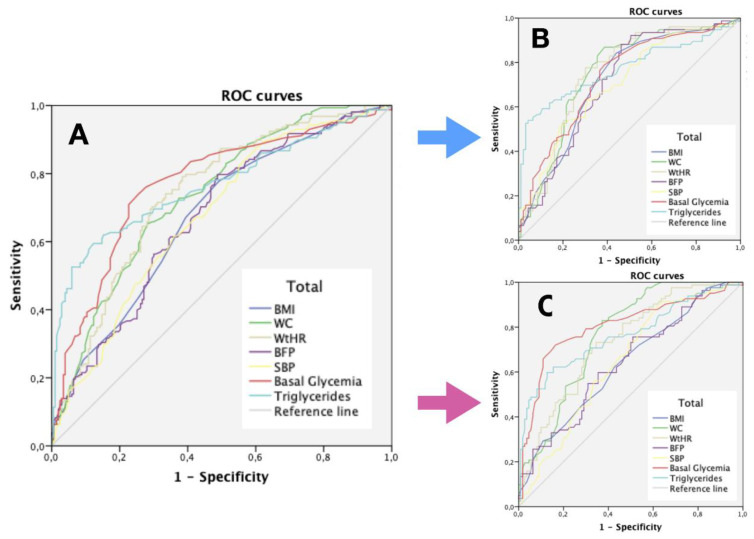
ROC curves for anthropometric and analytical variables predictive of MetS in the elderly (*n* = 158). (**A**) ROC curves of anthropometric and analytical variables predictive of MetS in the total sample (*n* = 361); (**B**) ROC curves of the most essential anthropometric and analytical variables of MetS in older men (*n* = 169); (**C**) ROC curves of the most essential anthropometric and analytical variables of MetS in older women (*n* = 198). *ROC: receiver operator characteristic; BMI: body mass index; WC: waist circumference; WtHR: waist height ratio; BFP: body fat percentage; SBP: systolic blood pressure.*

For predictive analysis of anthropometric variables, only those with OR crude with statistical significance were analyzed. Furthermore, only the variables with the best values of AUC (BMI, WC, WtHR and BFP) were presented. Figure 1A shows the different ROC curves for the anthropometric and analytical variables with the best AUC, separated according to sex in Figure 1B,C.

Table 2 shows the AUC, cutoff points, sensitivity (S), specificity (SP) and Youden index (J) for the most significant anthropometric and analytical variables. Within the analytical variables, basal glucose (AUC = 0.77; S = 74.1%, SP = 74.4%, J = 0.48) and triglycerides (AUC = 0.77; S = 59.5%, SP = 87.7%, J = 0.47) stand out. As for the anthropometric variables, the WHtR (AUC = 0.74; S = 79.1%, SP = 61.6%, J = 0.41) and the WC (AUC = 0.73; S = 65.2%, SP = 71.9%, J = 0.37) exhibited a higher predictive capacity. 

With regard to gender, in the case of men, the triglycerides (AUC = 0.77; S = 53.9%, SP = 95.7%, J = 0.50), the WC (AUC = 0.75; S = 86.8%, SP = 61.3%, J = 0.48) and the WHtR (AUC = 0.75; S = 77.6%, SP = 69.9%, J = 0.47) were prominent. However, for women, the best values were obtained for basal glucose (AUC = 0.80; S = 72%, SP = 83.6%, J = 0.56), triglycerides (AUC = 0.77; S = 59.8%, SP = 87.3%, J = 0.47) and the WC (AUC = 0.77; S = 82.9%, SP = 62.7%, J = 0.46).

### 3.3. Design of Decision Tree to Detect MetS Based on Non-Invasive or Minimally Invasive Tests 

The following step was to develop different decision trees for the detection of MetS by taking the predictive variables that showed the most significant diagnostic accuracy. Different decision trees were elaborated, placed for each of the independent variables as the first entry. Of all the trees obtained, those showing the best predictive capacity satisfied most of the following criteria: no independent variable placed first, a minimum number of 60 individuals in the parent node, and a minimum number of 30 subjects in the child node.

**Figure 2:**
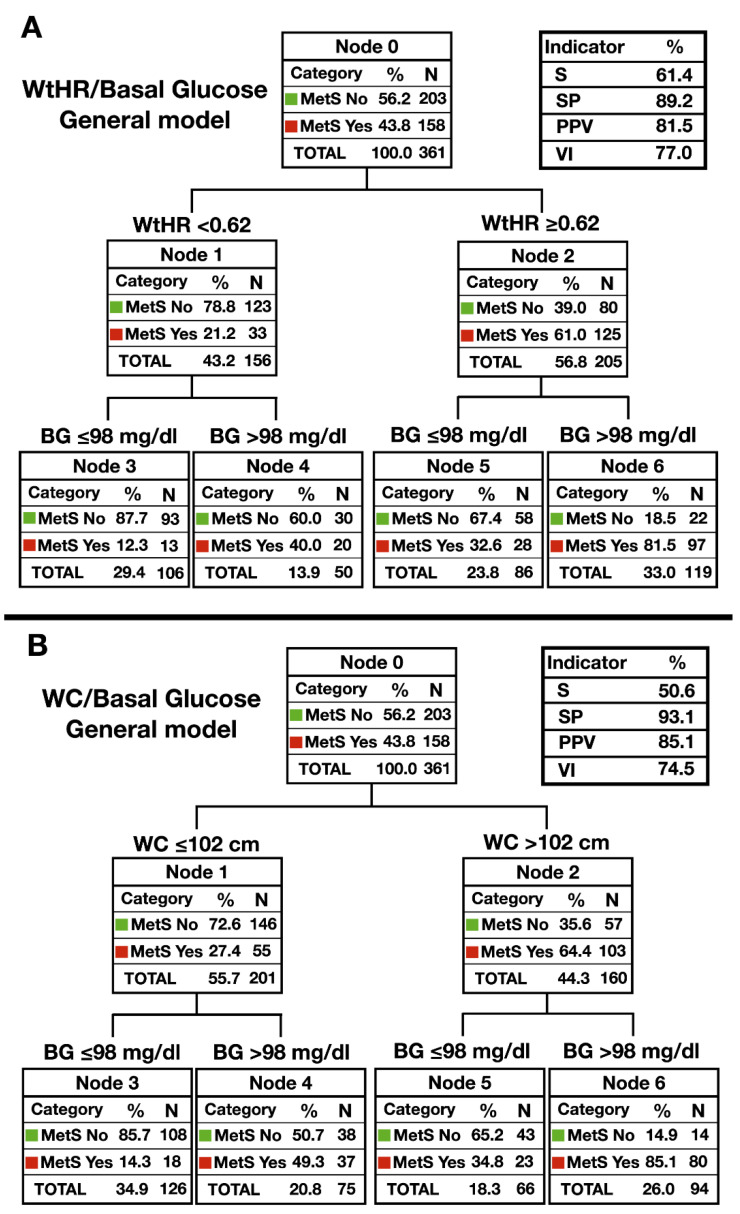
Diagnostic decision trees for detection of MetS in the elderly (*n* = 158). (**A**) Model that uses WtHR and basal glucose in the total sample; (**B**) Model that uses WC and basal glucose in the total sample. MetS: metabolic syndrome; WtHR: waist–height ratio, WC: waist circumference, BG: basal glucose. S: sensibility; SP: specificity; PPV: positive predictive value; VI: validity index.

Figure 2 shows the decision trees (classification algorithm) with the best predictive capacity. The best model for the overall sample Figure 2A was that formed by the WHtR and the basal glucose (S = 61.4%, SP = 89.2%, PPV = 81.5, validity index or VI = 77%); whereas the combination of WC and basal glucose offered a worse model in the overall sample, Figure 2B (S = 50.6%, SP = 93.1%, PPV = 85.1%, VI 74.5%).

**Figure 3:**
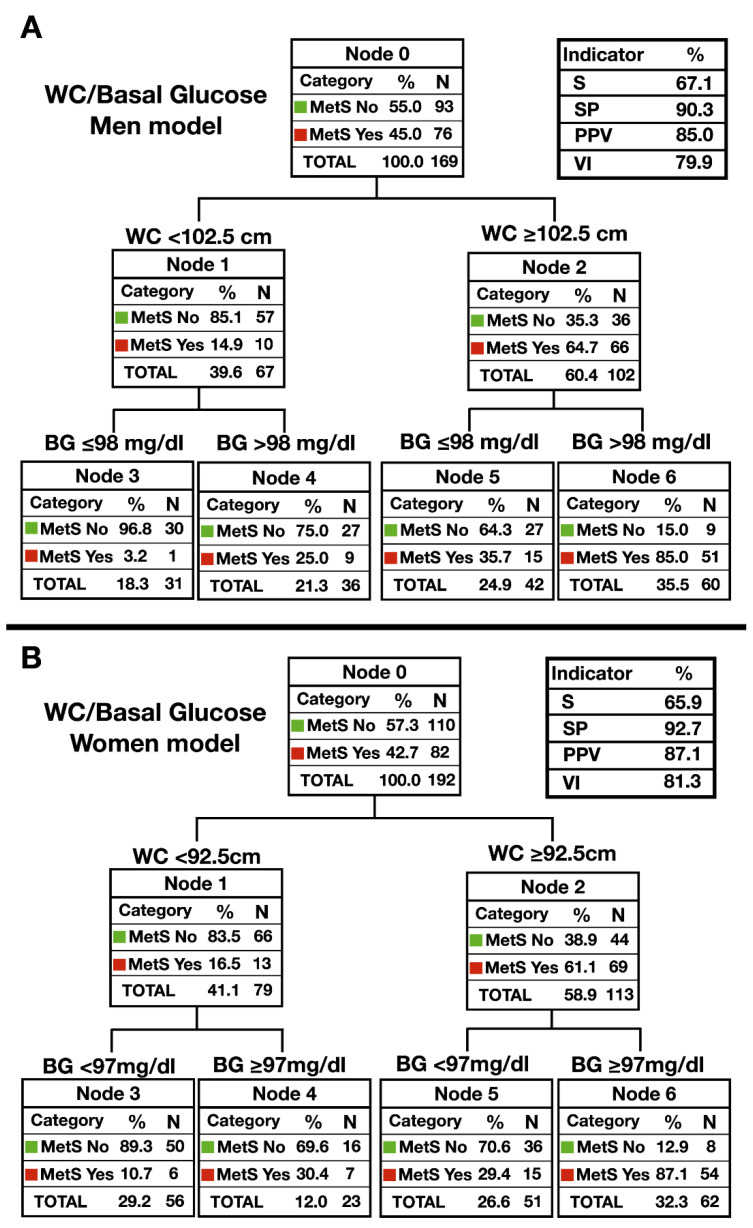
Diagnostic decision trees for detection of MetS in elderly women (*n* = 82) and men (*n* = 76). (**A**) Model that uses WC and basal glucose in older men; (**B**) Model that uses WC and basal glucose in older women. MetS: metabolic syndrome; WtHR: waist–height ratio, WC: waist circumference, BG: basal glucose. S: sensibility; SP: specificity; PPV: positive predictive value; VI: validity index.

Separating by gender Figure 3, the decision trees with the highest predictive capacity were obtained with the WC and the basal glucose. The one with the highest predictive capacity in men, Figure 3A, employed the WC cut-off points ≥ 102.5 cm and basal glucose > 98 mg/dL (S = 67.1%, SP = 90.3%, PPV = 85%, VI = 79.9%). Regarding women, Figure 3B, the cut-off points were established at WC ≥ 92.5 cm and in basal glucose at ≥ 97 mg/dL (S = 65.9%, SP = 92.7%, PPV = 87.1%, VI = 81.3%).

Table 3 shows the principal predictive indicators for the MetS diagnostic models (prevalence, sensitivity, specificity, positive predictive value, negative predictive value, positive and negative likelihood ratio, validity index, and Youden index) with their corresponding 95% confidence intervals. As a final decision tree, a specific model for men and women has been selected (J = 0.59), respectively, using the WC and the basal glucose, since the Youden index in both cases improves the diagnostic accuracy by 9% compared to the global tree.

## 4. Discussion

This work proposes a new minimally invasive method for the detection of MetS in the elderly population, based on a metabolic, partly anthropometric, phenotype, and defined by the WC and basal glucose. 

The general prevalence of the MetS in our study was 43.8%, which was similar to the data found in other MetS prevalence studies. In the ENRICA study (*n* = 2050 older people), MetS prevalence in the over 65s was estimated at around 42.3% [5]. In Brazil, different prevalence in older people were seen according to their age groups: 60–69 years (48.6%), 70–79 (41.6%), and 80 or over (45.2%) [21]. When differentiating between sexes, a prevalence of MetS is found of 45% in men and 42% in women, which differs from most studies in which the prevalences are significantly higher [3,22,23,24]. However, in a Mexican study [25] (*n* = 516), a higher prevalence in men, 75.7%, was shown than in women (70.4%).

The anthropometric variables that displayed the highest predictive capacity (larger AUC) were the WHtR (0.74) and the WC (0.73). Concerning gender, in the older men, their WHtR and WC stood out with an AUC of 0.75 for both. In the women, the best anthropometric variable was the WC, with an AUC of 0.77. All these results agreed with the scientific literature, in whose analyses of cardiovascular risk and metabolic syndrome in older people, both the WHtR and the WC, stood out over the rest in as far as their predictive power was concerned [26,27,28].

Regarding the analytical variables, basal glucose and the triglycerides presented an AUC of 0.77. Basal glucose achieved a greater sensitivity and specificity together (J = 0.48). Separately, in men, the triglycerides (AUC = 0.77) and basal glucose (AUC = 0.76) were notable, whereas in women it was basal glucose (AUC = 0.80) and the triglycerides (AUC = 0.76), with the best Youden Index being for basal glucose (J = 0.57). Thus, it was corroborated that the level of blood glucose is an analytical variable related to the MetS, due to the increase in the risk and mortality of cardiovascular disease [29], especially in older women [30]. However, in other works, the WC, blood pressure, and basal glucose were the variables that presented the highest prevalence of MetS in the elderly population [31].

After performing various tests for constructing the clinical decision tree by the CHAID method, up to four decision trees were selected. Finally, the best option for each sex was considered to be; the tree configured with the WC and basal glucose with validity indexes of 79.9% and 81.3%, for men and women, respectively. It can likewise be regarded as being a minimally invasive method since the basal glucose can be obtained from the basal capillary glucose. This test has already been validated as a screening method for the detection of type 2 diabetes or altered basal glucose [32].

The CHAID method has already proved to be a useful tool in the diagnosis of MetS [33]. In other works, some non- or minimally invasive methods have been proposed for the MetS. It should be noted that in the revised literature for MetS, we have not found publications with clinical decision trees constructed by the CHAID method that have reported negative evaluations or results. In Miller et al., a decision tree elaborated with CHAID was also used to detect MetS in a young adult population, simultaneously including anthropometry and analytical variables like WC and glucose, among others [34]. In a recent study, WHtR was reported as being a screening method for determining MetS in an elderly Japanese population [35]. The NIM-MetS method suggested by Romero-Sadaña, M et al. [36] is also worth noting; this method is based on the measurement of WHtR and blood pressure in a working population. In DeKroon et al.’s study, the decision tree considered three anthropometric variables (BMI, WC, and blood pressure) to identify the MetS in young adults. The risk of having the MetS was heavier when the WC and the blood pressure were high [37]. Finally, DY. Hsiung et al. recommended non-invasive variables (blood pressure, the BMI, heart rate) as risk predictors of MetS at its earliest stage [38].

The cut-off points for the anthropometric variables are specific for each population group (ethnicity, age, and gender) [3,23,24,39]. Although the WC cut-off point for men (102.5 cm) was similar to that used in the harmonized definition, in the WC/basal glucose model for older women, a different point from that established for the WC (92.5 cm) is observed. In a study in Brazil [40], with 113 older women, a cut-off MetS predictor point of 92 cm for WC was observed, whereas Akbulut et al. (*n* = 137) [41] found another one of 95.9 cm. This fact demonstrates the need, in our opinion, to reconsider whether the cut-off point proposed by the harmonized definition, IDF, and ATP-III of WC, should be adapted for older women. 

The model proposed here, both for men and women, are useful for assessing whether or not to take a blood test. It is an especially important aspect in situations with lower accessibility to health resources like rural populations that are highly dispersed geographically. If, for example, we have an older woman with a WC of 93 cm and a basal glucose of 99 mg/dL, she would be highly likely to have the MetS (VPP = 87.1%), so that it would be appropriate to perform a confirmatory analysis. If, on the contrary, we have the case of one with a WC of 91 cm and a basal glucose of 96 mg/dL, she would have a scant probability of suffering from the MetS (VPN = 78.5%), so that no analysis would be necessary.

The high PPV’s of the models cited agree with a study of hospitalized older patients, in which WC was proposed as an anthropometric measurement for improving the PPV for the MetS diagnosis [42]. Besides, both models presented a higher specificity (90.3% men, 92.7% men) and a lower sensitivity (67.1% men, 65.9% women); whereas the positive predictive values of the models were PPV: 85% and NPV: 77.1%, for men; and PPV: 87.1% and NPV: 78.5%, for women. With all these data, we can highlight that a test with a high specificity would be beneficial for the diagnosis of this cluster of cardiovascular risk factors, which have a high prevalence, such as the metabolic syndrome in the elderly. If the test is negative, further invasive testing can be avoided, saving costs and unnecessary discomfort for our patients.

### Limitations

The CHAID methodology used for setting up the decision tree recommends large sample sizes in order to optimize their statistical significance. The sample taken in this study, separated per sexes, was for men (*n* = 198) and women (*n* = 169). The criteria for forming parent and child nodes were moderate ones (60 and 30, respectively). It would, therefore, be helpful to employ larger samples for future studies to obtain a higher reliability.

It would also be of interest to make a study to validate the simplified method proposed using the same reference test (harmonized definition of the MetS) in another sample. It would permit us to find out the diagnostic validity indicators of the models proposed according to gender, in another population, and in a different clinical context from the one that was used to develop this present predictive model.

## 5. Conclusions

The simplified MetS diagnostic method in older people proposed using the measurement of the WC and taking basal capillary glucose is seen to be a highly accurate one for men (VI = 79.9%) and women (VI = 81.3%).

It is a minimally invasive diagnostic method with numerous advantages to others. First, it only uses two variables (WC and basal capillary glucose) that are easily accessible in any healthcare context (i.e., primary and hospital attention, occupational health, nursing homes), which reduces the frequency of using blood tests and only in those cases requiring a confirmation. Finally, it is an economical, versatile method and is easily interpreted through the algorithms proposed.

Incidentally, it was observed that a WC starting from 92.5 cm performs better as a MetS predictor cut-off point in older women than that proposed in the IDF definitions, harmonized and ATP-III.

## Figures and Tables

**Table 1:** Characteristics and logistic regression of the elderly population (*n* = 361) with or without MetS.

Variables		Total (*N* = 361)	MetS Yes (*N* = 158)	MetS No (*N* = 203)	OR Crude (CI 95%)	*p-*Value
Age (years)		73.2 (6.4)	73.2 (6.0)	73.3 (6.7)	1.00 (0.97–1.03)	NS
Gender	Women	192 (53.2%)	82 (42.7%)	110 (57.3%)	1	-
	Men	169 (46.8%)	76 (45.0%)	93 (55.0%)	1.10 (0.72–1.66)	NS
Education level	N/P	303 (83.9%)	129 (42.6%)	174 (57.4%)	1	-
	S/U	58 (16.1%)	29 (50%)	29 (50%)	1.35 (0.77–2.37)	NS
Smoking	Non-smoker	257 (71.2%)	108 (42%)	149 (58%)	1	
	Quitter	78 (21.6%)	36 (46.2%)	42 (53.8%)	1.18 (0.71–1.97)	NS
	Smoker	26 (7.2%)	14 (53.8%)	12 (46.2%)	1.61 (0.72–3.62)	NS
Risk drinker	No	286 (79.2%)	124 (43.4%)	162 (56.6%)	1	-
	Yes	75 (20.8%)	34 (45.3%)	41 (54.7%)	1.08 (0.65–1.81)	NS
Physical activity	No	235 (65.1%)	110 (46.8%)	125 (53.2%)	1	-
	Yes	126 (34.9%)	48 (38.1%)	78 (61.9%)	0.70 (0.45–1.09)	NS
Fruit/vegetables consumption	No	269 (74.5%)	104 (38.7%)	165 (61.3%)	1	-
	Yes	92 (25.5%)	54 (58.7%)	38 (41.3%)	0.44 (0.27–0.72)	<0.01
Polymedicated	No	270 (74.8%)	104 (38.5%)	166 (61.5%)	1	-
	Yes	91 (25.2%)	54 (59.3%)	37 (40.7%)	2.33 (1.43–3.78)	<0.01
Anthropometric variables	WC (cm)	100.41 (12.2)	105.9 (10.5)	96.1 (11.6)	1.08 (1.06–1.11)	<0.001
	HC (cm)	106.72 (9.50)	109.41 (9.60)	104.63 (8.92)	1.06 (1.03–1.08)	<0.001
	WHR *	0.94 (0.09)	0.97 (0.08)	0.92 (0.09)	1.18 (1.10–1.26)	<0.001
	WHtR *	0.64 (0.07)	0.67 (0.06)	0.61 (0.06)	1.29 (1.83–1.41)	<0.001
	BFP (%)	35.6 (5.3)	37.5 (5.4)	34.2 (4.8)	1.13 (1.08–1.19	<0.001
	ABSI *	0.13 (0.01)	0.14 (0.01)	0.13 (0.01)	0.35 (0.21–0.60)	<0.001
	BAI	36.15 (6.71)	37.10 (6.83)	35.40 (6.53)	1.04 (1.01–1.07)	<0.05
	BMI (kg/m^2^)	29.2 (4.5)	30.7 (4.6)	28.0 (4.0)	1.16 (1.10–1.23)	<0.001
	SBP (mmHg)	140.2 (19.9)	146.8 (18.4)	135.1 (19.53)	1.03 (1.02–1.05)	<0.001
	DBP (mmHg)	77.5 (10.6)	78.5 (11.1)	76.7 (10.1)	1.02 (1–1.04)	NS
Biochemicalvariables	BG (mg/dL)	105.4 (31.3)	118.4 (38.5)	95.3 (19.0)	1.04 (1.03–1.05)	<0.001
	cHDL (mg/dL)	55.3 (14.7)	49.5 (12.9)	59.9 (14.4)	0.94 (0.92–0.96)	<0.001
	cLDL (mg/dL)	121.6 (35.7)	115.0 (37.1)	126.7 (33.7)	0.99 (0.98–0.997)	<0.01
	TG (mg/dL)	123.9 (56.9)	152.8 (67.3)	101.5 (33.1)	1.02 (1.02–1.03)	<0.001

MetS: metabolic syndrome; CI: confidence interval; NS: not significant; N/P: None or Primary Studies; S/U: Secondary or University studies; WC: waist circumference; HC: hip circumference; WHR: waist–hip ratio; WHtR: waist–height ratio; BFP: body fat percentage; ABSI: A Body Shape Index; BAI: Body Adiposity Index; BMI: body mass index; SBP: systolic blood pressure; DBP: diastolic blood pressure; BG: basal glucose; cHDL: cholesterol high-density lipoprotein; cLDL: cholesterol low-density lipoprotein TG: triglycerides. * WHR, WHtR, and ABSI: transformed as the square root of the Napierian Logarithm, only for logistic regression analysis.

**Table 2:** Diagnostic accuracy of predictive variables for MetS in the elderly population (*n* = 158). Total sample and according to gender.

Gender	Variable	AUC	*p*-Value	95% CI	Cut-Off Point	S (%)	SP (%)	J
Total	BG (mg/dL)	0.77	<0.001	0.72–0.82	98.5	74.1	74.4	0.48
	TG (mg/dL)	0.77	<0.001	0.71–0.82	137	59.5	87.7	0.47
	WHtR	0.74	<0.001	0.69–0.79	0.62	79.1	61.6	0.41
	WC (cm)	0.73	<0.001	0.68–0.78	102.1	65.2	71.9	0.37
	BMI (kg/m^2^)	0.67	<0.001	0.62–0.73	27.5	77.8	50.7	0.29
	SBP (mmHg)	0.67	<0.001	0.62–0.73	129.5	89.2	37.9	0.27
	Body fat (%)	0.67	<0.001	0.62–0.73	33.6	79.7	51.2	0.31
Men	BG (mg/dL)	0.73	<0.001	0.66–0.81	98.5	78.9	61.3	0.40
	TG (mg/dL)	0.77	<0.001	0.69–84.5	145.5	53.9	95.7	0.50
	WHtR	0.75	<0.001	0.68–0.83	0.64	77.6	69.9	0.47
	WC (cm)	0.75	<0.001	0.68–0.83	102.75	86.8	61.3	0.48
	BMI (kg/m^2^)	0.72	<0.001	0.64–0.80	27.5	84.2	55.9	0.40
	SBP (mmHg)	0.71	<0.001	0.63–0.78	141.5	63.2	68.8	0.32
	Body fat (%)	0.71	<0.001	0.63–0.79	33	88.2	53.8	0.42
Women	BG (mg/dL)	0.80	<0.001	0.74–0.87	97.5	72	83.6	0.56
	TG (mg/dL)	0.77	<0.001	0.69–0.83	138.5	59.8	87.3	0.47
	WHtR	0.73	<0.001	0.67–0.81	0.62	73.2	65.5	0.39
	WC (cm)	0.77	<0.001	0.70–0.83	93.25	82.9	62.7	0.46
	BMI (kg/m^2^)	0.64	<0.001	0.56–0.72	28.5	62.2	57.3	0.19
	SBP (mmHg)	0.65	<0.001	0.57–0.72	129.5	90.2	62.7	0.27
	Body fat (%)	0.64	<0.001	0.56–0.72	33.7	75.6	49.1	0.25

ROC: receiver operator characteristic; AUC: Area under the curve; S: sensibility, SP: specificity, J: Youden index, MetS: metabolic syndrome, BG: basal glucose; TG: triglycerides; WHtR: waist–height ratio, WC: waist circumference, BMI: body mass index SBP: systolic blood pressure.

**Table 3:** Leading predictive indicators for the MetS diagnosis models in the elderly population.

Indicator	Total Percentage (IC95%)		Men Percentage (IC 95%)	Women Percentage (IC 95%)
	WHtR/BG	WC/BG	WC/BG	WC/BG
Prevalence	43.8% (38.6–49.1)		45% (37.3–52.8)	42.7% (35.6–50)
Sensibility	61.4% (53.6–68.6)	50.6% (42.9–58.3)	67.1% (55.9–76.6)	65.9% (55.1–75.2)
Specificity	89.2% (84.1–92.7)	93.1% (88.8–95-8)	90.3% (82.6–94.8)	92.7% (86.3–96.3)
PPV	81.5% (73.6–87.5)	85.1% (76.5–90.9)	85% (73.9–91.9)	87.1% (76.6–93.3)
NPV	74.8% (69–79.8)	70.8% (65.1–75.9)	77.1% (68.3–84)	78.5% (70.6–84.7)
LH+	5.66 (3.75–8.57)	7.34 (4.33–12.45)	6.93 (3.65–13.16)	9.05 (4.56–17.97)
LH-	0.43 (0.35–0.53)	0.53 (0.45–0.63)	0.36 (0.26–051)	0.37 (0.27–0.50)
VI	77% (72.4–81.1)	74.5% (69.8–78.7)	79.9% (73.2–85.2)	81.3% (75.1–86.1)
J	0.51 (0.42–0.59)	0.44 (0.35–052)	0.57 (0.45–0.7)	0.59 (0.47–0.7)

PPV: positive predictive value; NPV: negative predictive value; LH+: likelihood ratio positive; LH-: likelihood ratio negative. VI: validity Index; J: Youden’s Index; WHtR: waist–height ratio, WC: waist circumference, BG: basal glucose.

## References

[1] Zimmet P.Z., Alberti K.G.M.M., Shaw J.E. (2005). Mainstreaming the Metabolic Syndrome: A Definitive Definition. Med. J. Aust..

[2] Salminen M., Kuoppamäki M., Vahlberg T., Räihä I., Irjala K., Kivelä S.-L. (2013). Metabolic Syndrome Defined by Modified International Diabetes Federation Criteria and Type 2 Diabetes Mellitus Risk: A 9-Year Follow-up among the Aged in Finland. Diab. Vasc. Dis. Res..

[3] Aguilar M., Bhuket T., Torres S., Liu B., Wong R.J. (2015). Prevalence of the Metabolic Syndrome in the United States, 2003-2012. JAMA.

[4] Yen Y.-F., Hu H.-Y., Lin I.-F., Lai Y.-J., Su V.Y.-F., Pan S.-W., Ting W.-Y., Su W.-J. (2015). Associations of Metabolic Syndrome and Its Components with Mortality in the Elderly: A Cohort Study of 73,547 Taiwanese Adults. Medicine (Baltimore).

[5] Guallar-Castillón P., Pérez R.F., López García E., León-Muñoz L.M., Aguilera M.T., Graciani A., Gutiérrez-Fisac J.L., Banegas J.R., Rodríguez-Artalejo F. (2014). Magnitude and Management of Metabolic Syndrome in Spain in 2008-2010: The ENRICA Study. Rev. Esp. Cardiol. (Engl. Ed.).

[6] Simons L.A., Simons J., Friedlander Y., McCallum J. (2007). Does a Diagnosis of the Metabolic Syndrome Provide Additional Prediction of Cardiovascular Disease and Total Mortality in the Elderly? The Dubbo Study. Med. J. Aust..

[7] Sagun G., Oguz A., Karagoz E., Filizer A.T., Tamer G., Mesci B. (2014). Application of Alternative Anthropometric Measurements to Predict Metabolic Syndrome. Clinics (Sao Paulo).

[8] Liu P., Ma F., Lou H., Liu Y. (2013). The Utility of Fat Mass Index vs. Body Mass Index and Percentage of Body Fat in the Screening of Metabolic Syndrome. BMC Public Health.

[9] Pischon T., Boeing H., Hoffmann K., Bergmann M., Schulze M.B., Overvad K., van der Schouw Y.T., Spencer E., Moons K.G.M., Tjønneland A. (2008). General and Abdominal Adiposity and Risk of Death in Europe. N. Engl. J. Med..

[10] Cornier M.-A., Després J.-P., Davis N., Grossniklaus D.A., Klein S., Lamarche B., Lopez-Jimenez F., Rao G., St-Onge M.-P., Towfighi A. (2011). Assessing Adiposity: A Scientific Statement from the American Heart Association. Circulation.

[11] MacKay M.F., Haffner S.M., Wagenknecht L.E., D’Agostino R.B., Hanley A.J.G. (2009). Prediction of Type 2 Diabetes Using Alternate Anthropometric Measures in a Multi-Ethnic Cohort: The Insulin Resistance Atherosclerosis Study. Diabetes Care.

[12] Alberti K.G.M.M., Eckel R.H., Grundy S.M., Zimmet P.Z., Cleeman J.I., Donato K.A., Fruchart J.-C., James W.P.T., Loria C.M., Smith S.C. (2009). Harmonizing the Metabolic Syndrome: A Joint Interim Statement of the International Diabetes Federation Task Force on Epidemiology and Prevention; National Heart, Lung, and Blood Institute; American Heart Association; World Heart Federation; International Atherosclerosis Society; and International Association for the Study of Obesity. Circulation.

[13] López T.M., Camacho M.O.C., Morgado D.P., López Rubio S., Domínguez Camacho J.C., Morales Serna J.C. (2012). Prevalencia de Polimedicación y Riesgo Vascular En La Población Mayor de 65 Años. Atención Primaria.

[14] Soriguer F., Valdés S., Tapia M.J., Esteva I., Ruiz de Adana M.S., Almaraz M.C., Morcillo S., García Fuentes E., Rodríguez F., Rojo-Martinez G. (2012). [Validation of the FINDRISC (FINnish Diabetes RIsk SCore) for prediction of the risk of type 2 diabetes in a population of southern Spain. Pizarra Study]. Med. Clin. (Barc.).

[15] Babor T.F., Higgins-Biddle J., Saunders J., Monteiro M.G. (2001). AUDIT: The Alcohol Use Disor- Ders Identi Cation Test. Guidelines for Use in Primary Care.

[16] Lohman T.G., Roche A.F., Martorell R. (1988). Anthropometric Standardization Reference Manual.

[17] (2019). Guía ESC/ESH 2018 Sobre el diagnóstico y tratamiento de la hipertensión arterial. Rev. Esp. Cardiol..

[18] Bergman R.N., Stefanovski D., Buchanan T.A., Sumner A.E., Reynolds J.C., Sebring N.G., Xiang A.H., Watanabe R.M. (2011). A Better Index of Body Adiposity. Obesity (Silver Spring).

[19] Krakauer N.Y., Krakauer J.C. (2012). A New Body Shape Index Predicts Mortality Hazard Independently of Body Mass Index. PLoS ONE.

[20] General Assembly of the World Medical Association (2014). World Medical Association Declaration of Helsinki: Ethical Principles for Medical Research Involving Human Subjects. J. Am. Coll. Dent..

[21] Saad M.A.N., Cardoso G.P., de Martins W.A., Velarde L.G.C., Filho C., Da R.A., Saad M.A.N., Cardoso G.P., de Martins W.A., Velarde L.G.C. (2014). Prevalence of Metabolic Syndrome in Elderly and Agreement among Four Diagnostic Criteria. Arquivos Brasileiros de Cardiologia.

[22] Maggi S., Noale M., Gallina P., Bianchi D., Marzari C., Limongi F., Crepaldi G. (2006). Metabolic Syndrome, Diabetes, and Cardiovascular Disease in an Elderly Caucasian Cohort: The Italian Longitudinal Study on Aging. J. Gerontol. A Biol. Sci. Med. Sci..

[23] Orces C.H., Gavilanez E.L. (2017). The Prevalence of Metabolic Syndrome among Older Adults in Ecuador: Results of the SABE Survey. Diabetes Metab. Syndr. Clin. Res. Rev..

[24] Li W., Song F., Wang X., Wang L., Wang D., Yin X., Cao S., Gong Y., Yue W., Yan F. (2018). Prevalence of Metabolic Syndrome among Middle-Aged and Elderly Adults in China: Current Status and Temporal Trends. Ann. Med..

[25] Ortiz-Rodríguez M.A., Yáñez-Velasco L., Carnevale A., Romero-Hidalgo S., Bernal D., Aguilar-Salinas C., Rojas R., Villa A., Tur J.A. (2017). Prevalence of Metabolic Syndrome among Elderly Mexicans. Arch. Gerontol. Geriatr..

[26] Guasch-Ferré M., Bulló M., Martínez-González M.Á., Corella D., Estruch R., Covas M.-I., Arós F., Wärnberg J., Fiol M., Lapetra J. (2012). Waist-to-Height Ratio and Cardiovascular Risk Factors in Elderly Individuals at High Cardiovascular Risk. PLoS ONE.

[27] Zeng Q., He Y., Dong S., Zhao X., Chen Z., Song Z., Chang G., Yang F., Wang Y. (2014). Optimal Cut-off Values of BMI, Waist Circumference and Waist:Height Ratio for Defining Obesity in Chinese Adults. Br. J. Nutr..

[28] Motala A.A., Esterhuizen T., Pirie F.J., Omar M.A.K. (2011). The Prevalence of Metabolic Syndrome and Determination of the Optimal Waist Circumference Cut-off Points in a Rural South African Community. Diabetes Care.

[29] Wang A., Liu X., Xu J., Han X., Su Z., Chen S., Zhang N., Wu S., Wang Y., Wang Y. (2017). Visit-to-Visit Variability of Fasting Plasma Glucose and the Risk of Cardiovascular Disease and All-Cause Mortality in the General Population. J. Am. Heart Assoc..

[30] Hsiao F.-C., Hsieh C.-H., Wu C.-Z., Hsu C.-H., Lin J.-D., Lee T.-I., Pei D., Chen Y.-L. (2013). Elevated Fasting Glucose Levels within Normal Range Are Associated with an Increased Risk of Metabolic Syndrome in Older Women. Eur. J. Intern. Med..

[31] Moebus S., Balijepalli C., Lösch C., Göres L., von Stritzky B., Bramlage P., Wasem J., Jöckel K.-H. (2010). Age- and Sex-Specific Prevalence and Ten-Year Risk for Cardiovascular Disease of All 16 Risk Factor Combinations of the Metabolic Syndrome—A Cross-Sectional Study. Cardiovasc. Diabetol..

[32] Kruijshoop M., Feskens E.J.M., Blaak E.E., de Bruin T.W.A. (2004). Validation of Capillary Glucose Measurements to Detect Glucose Intolerance or Type 2 Diabetes Mellitus in the General Population. Clin. Chim. Acta.

[33] Worachartcheewan A., Nantasenamat C., Isarankura-Na-Ayudhya C., Pidetcha P., Prachayasittikul V. (2010). Identification of Metabolic Syndrome Using Decision Tree Analysis. Diabetes Res. Clin. Pract..

[34] Miller B., Fridline M., Liu P.-Y., Marino D. (2014). Use of CHAID Decision Trees to Formulate Pathways for the Early Detection of Metabolic Syndrome in Young Adults. Comput. Math. Methods Med..

[35] Kawamoto R., Kikuchi A., Akase T., Ninomiya D., Kumagi T. (2019). Usefulness of Waist-to-Height Ratio in Screening Incident Metabolic Syndrome among Japanese Community-Dwelling Elderly Individuals. PLoS ONE.

[36] Romero-Saldaña M., Fuentes-Jiménez F.J., Vaquero-Abellán M., Álvarez-Fernández C., Molina-Recio G., López-Miranda J. (2016). New Non-Invasive Method for Early Detection of Metabolic Syndrome in the Working Population. Eur. J. Cardiovasc. Nurs..

[37] de Kroon M.L.A., Renders C.M., Kuipers E.C.C., van Wouwe J.P., van Buuren S., de Jonge G.A., Hirasing R.A. (2008). Identifying Metabolic Syndrome without Blood Tests in Young Adults--the Terneuzen Birth Cohort. Eur. J. Public Health.

[38] Hsiung D.-Y., Liu C.-W., Cheng P.-C., Ma W.-F. (2015). Using Non-Invasive Assessment Methods to Predict the Risk of Metabolic Syndrome. Appl. Nurs. Res..

[39] Ravaglia G., Forti P., Maioli F., Bastagli L., Chiappelli M., Montesi F., Bolondi L., Patterson C. (2006). Metabolic Syndrome: Prevalence and Prediction of Mortality in Elderly Individuals. Diabetes Care.

[40] de Paula H.A.A., de Ribeiro R.C.L., de Rosado L.E.F.P.L., Abranches M.V., do Franceschini S.C.C. (2012). Classic Anthropometric and Body Composition Indicators Can Predict Risk of Metabolic Syndrome in Elderly. Ann. Nutr. Metab..

[41] Akbulut G., Köksal E., Bilici S., Acar Tek N., Yildiran H., Karadag M.G., Sanlier N. (2011). Metabolic Syndrome (MS) in Elderly: A Cross Sectional Survey. Arch. Gerontol. Geriatr..

[42] Patel P.V., Gilski D., Morrison J. (2010). Using Waist Circumference to Screen for Metabolic Syndrome in an Inpatient Population. Crit. Pathw. Cardiol..

